# Dataset on *in-vitro* study of chitosan-graphene oxide films for regenerative medicine

**DOI:** 10.1016/j.dib.2021.107472

**Published:** 2021-10-12

**Authors:** Ana Maria Valencia, Carlos Humberto Valencia, Fabio Zuluaga, Carlos David Grande-Tovar

**Affiliations:** a Grupo de investigación SIMERQO polímeros, Departamento de Química, Universidad del Valle, Calle 13 # 100-00, Cali 76001, Colombia; b Escuela de Odontología, Grupo Biomateriales dentales, Universidad del Valle, Calle 4B No. 36-00, Cali 76001, Colombia; c Grupo de investigación de Fotoquímica y Fotobiología, Universidad del Atlántico, Carrera 30 Número 8-49, Puerto Colombia, 081008, Colombia

**Keywords:** Chitosan, Graphene Oxide, Biocompatibility, Cell growth test, Regenerative medicine

## Abstract

Chitosan (CS) is well-known for its biological properties, especially its ability to induce tissue cicatrization. However, considerable research proved that CS presents a high inflammatory response and poor mechanical properties. For these reasons, we decided to use chitosan (CS) functionalized GO by a covalent bond (CS-GO). Due to the resistant structure of the GO and the high presence of oxygen functional groups on it, it will enhance the biocompatibility of the material. The data obtained in this investigation aimed to prove the possible application of CS-GO in regenerative medicine. For this reason, it was performed an *In vitro* analysis using brine shrimp to prove materials biocompatibility and gingival fibroblasts for a cell growth test.

## Specifications Table

**Table:** 

Subject	Biomaterials
Specific subject area	Biomaterials applied in tissue engineering
Type of data	Tables Images
How data were acquired	CS and CS-GO molecular weight were measured by Gel Permeation Chromatography (GPC) according to the USP guide, using two columns Shodex Ohpak 805 and 806, with many theoretical plates < 10,000. The measurements were carried out in an Agilent Infinity 1260 (Agilent Technologies, Santa Clara, California, USA) with a refractive index detector. For the biocompatibility assay, brine shrimp eggs were culture into a box with culture wells, a membrane of CS-GO, and the physical mix of CS and GO (CS/GO) were used in some wells, and living larvae were counted at 24, 36 and 48 h. For the growth cell test, a Leica inverted field microscope (Leica Microsystems, Mannheim, Germany) took images and a Neubauer camera (Blood Counting Chambers, BOECO, Hamburg Germany) to count cells.
Data format	Raw Analyzed
Parameters for data collection	For molecular weight measurements, it was necessary to dissolve samples into the mobile phase in a 1 mg/mL concentration and then filter it with a PVDF membrane of 0.45 µm. The parameters to which GPC analysis was carried out were a rate of 0.5 mL/min, a column temperature of 35 °C, and a sample volume of 20 µL. For the biocompatibility test, the Rocha-Filho protocol [1] was followed. For this, brine shrimp eggs were hydrated with seawater and cultured in a box with wells for cell culture. For the test, it was necessary membranes of CS-GO and CS/GO, one membrane was located in the well, and the biocompatibility was determined by counting living larvae. For the growth cell test, unfreeze cells were taken to a New Brunswick Galaxy 48R incubator with a complete culture medium. Eight membranes of CS-GO were deposited in a box with 12 culture wells with approximately 30,000 cells. After nine days, cells were detached, followed images were taken using a Leica inverted field microscope to verify cell growth. Finally, the number of cells was counted using a Neubauer camera.
Description of data collection	Cell images were taken using a Leica inverted field microscope, and a Neubauer camera obtained the number of cells.
Data source location	Universidad del Valle Cali, Valle del Cauca, Colombia Gingival fibroblasts were obtained from the cell bank of the *in-vitro* Cell Culture Laboratory of the Universidad del Valle in Cali, Colombia.
Data accessibility	With the article
Related research article	Valencia, A. M.; Valencia, C. H.; Zuluaga, F.; Grande-Tovar, C. D. Synthesis and Fabrication of Films Including Graphene Oxide Functionalized with Chitosan for Regenerative Medicine Applications. Heliyon **2021**, 7 (5), e07058. 10.1016/j.heliyon.2021.e07058.

**Value of the Data**

• “The data provided in this research analysis CS-GO cell biocompatibility and complement In vivo studies. It suggests the application of the material in tissue regeneration”.
• “The data presented here may be helpful to researchers who are performing research where biocompatibility of chitosan-grafted graphene oxide is needed”.
• “Information provided in this study can be used for in vitro and in vivo assays of chitosan of low molecular weight grafted with graphene oxide”.

## 1 Data Description

This dataset contains insightful information about how the molecular weight (MW) of chitosan is measured and how biocompatibility in vitro tests was carried out for research. CS and GO have been highly used for scaffolds, and the biocompatibility effect of CS-GO plays an essential role in tissular regeneration. Valencia et al. (2021) mentioned GO improved the mechanical properties of CS by its functionalization [2].

**Fig. 1:**
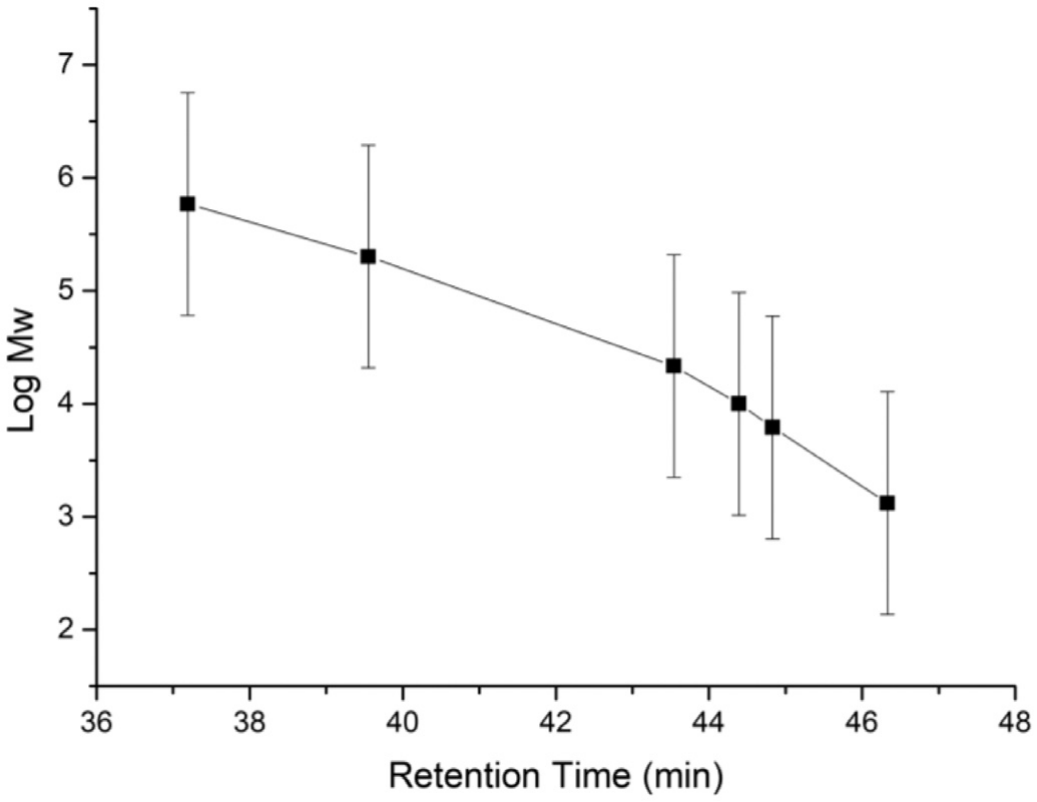
Calibration curve of GPC analysis with pullulan standards.

It was necessary to measure the product's molecular weight (Fig. 1 and Table 1) to prove the functionalization of CS with the GO. For this, a GPC analysis was carried out. Equipment was calibrated using six pullulan standards with a molecular weight of 1320-586800 Da.

**Table 1:** Molecular weight obtained by GPC analysis.

Sample	Mn (Da)	Mw (Da)
CS	2797	15,323
CS-GO	329,960	1291

Once the equipment was calibrated, it was possible to determine the molecular weight of the samples.

A biocompatibility test was carried out by a sample film (CS-GO and CS/GO) into a culture well, then seawater was added, and finally the brine shrimp larvae.

**Fig. 2:**
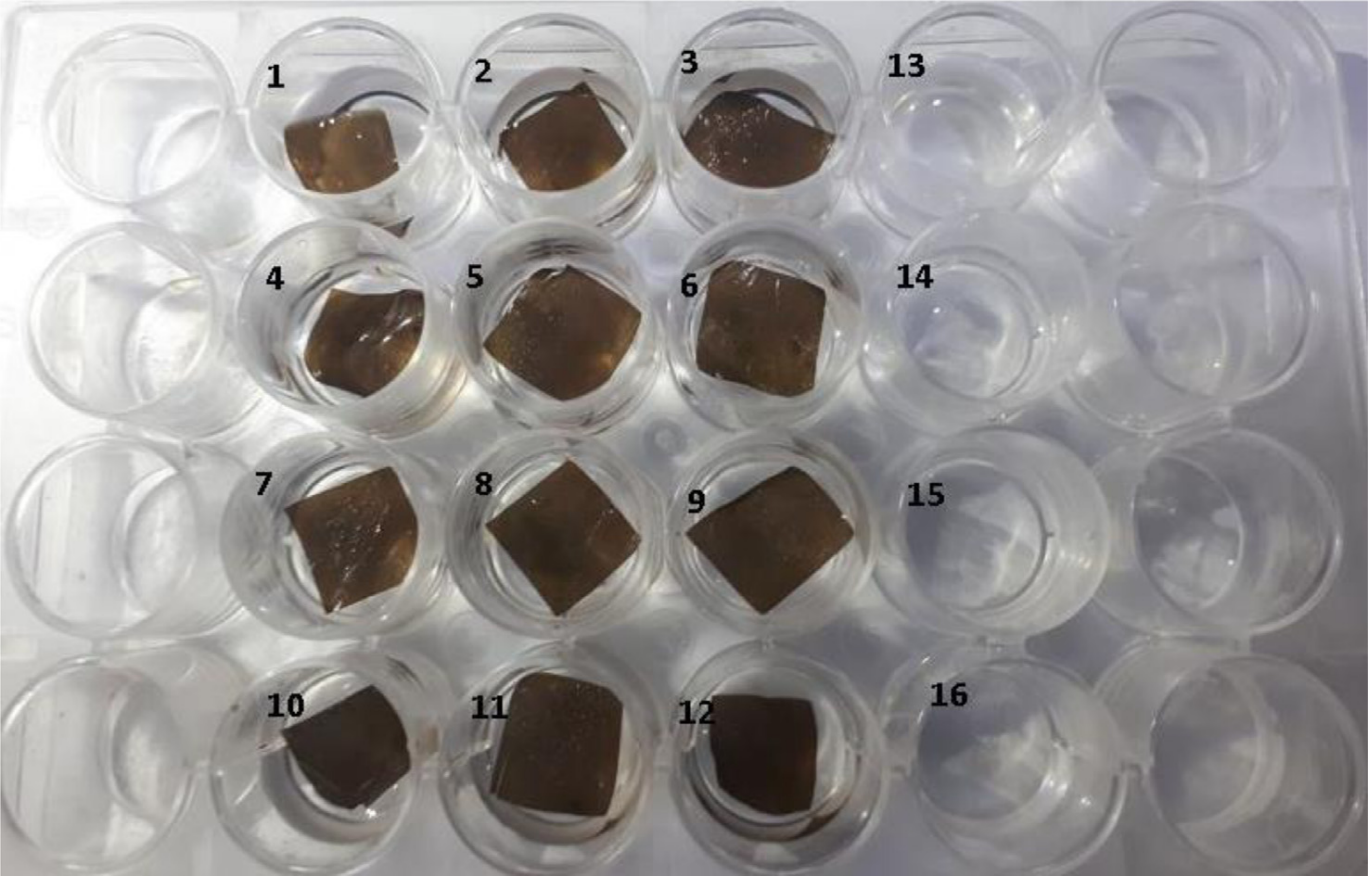
CS-GO and CS/GO films in seawater solution with brine shrimp. Culture wells 1–6, CS-GO samples. Culture wells 7–12, CS/GO samples, and culture wells 13–16, control with no pieces.

The biocompatibility was determined by counting live larvae at 24, 36, and 48 h (Fig. 2 and Table 2). The percentage of cytotoxicity was determined by the formula [3]:

(1) $$Cytotoxicity=\;\frac{x_{o}-x_{f}}{x_{T}}\times \;100\%$$

Where: $x_{o}=Initial\;live\;larvae,\;x_{f}=Final\;live\;larvae,\;x_{T}=Total\;larvae$

Then: x≥30%→IsnotBiocompatible

**Table 2:** Culture well results for brine shrimp tests.

CS/GO Sample (culture well ubication)	Death larvae	CS-GO Sample (culture well ubication)	Death larvae	Control-No sample (culture well ubication)	Death larvae
1	1	7	1	13	2
2	1	8	3	4	2
3	2	9	2	15	2
4	2	10	2	16	2
5	3	11	1	17	1
6	1	12	2	18	2
*Total live larvae*	50	*Total live larvae*	49	*Total live larvae*	49
*Average per group*	2±0.8	*Average per group*	2 ± 0.7	*Average per group*	2 ± 0.4

A death percentage equal to or greater than 30% allows the material to be considered toxic or non-biocompatible [4].

Toxicity percentage was obtained using Eq. (1):

CS-GO group: $Toxicity=\frac{60-49}{60}\times \;100\%=18\%$
CS/GO group: $\frac{60-50}{60}\times \;100\%=16\%$

**Fig. 3:**
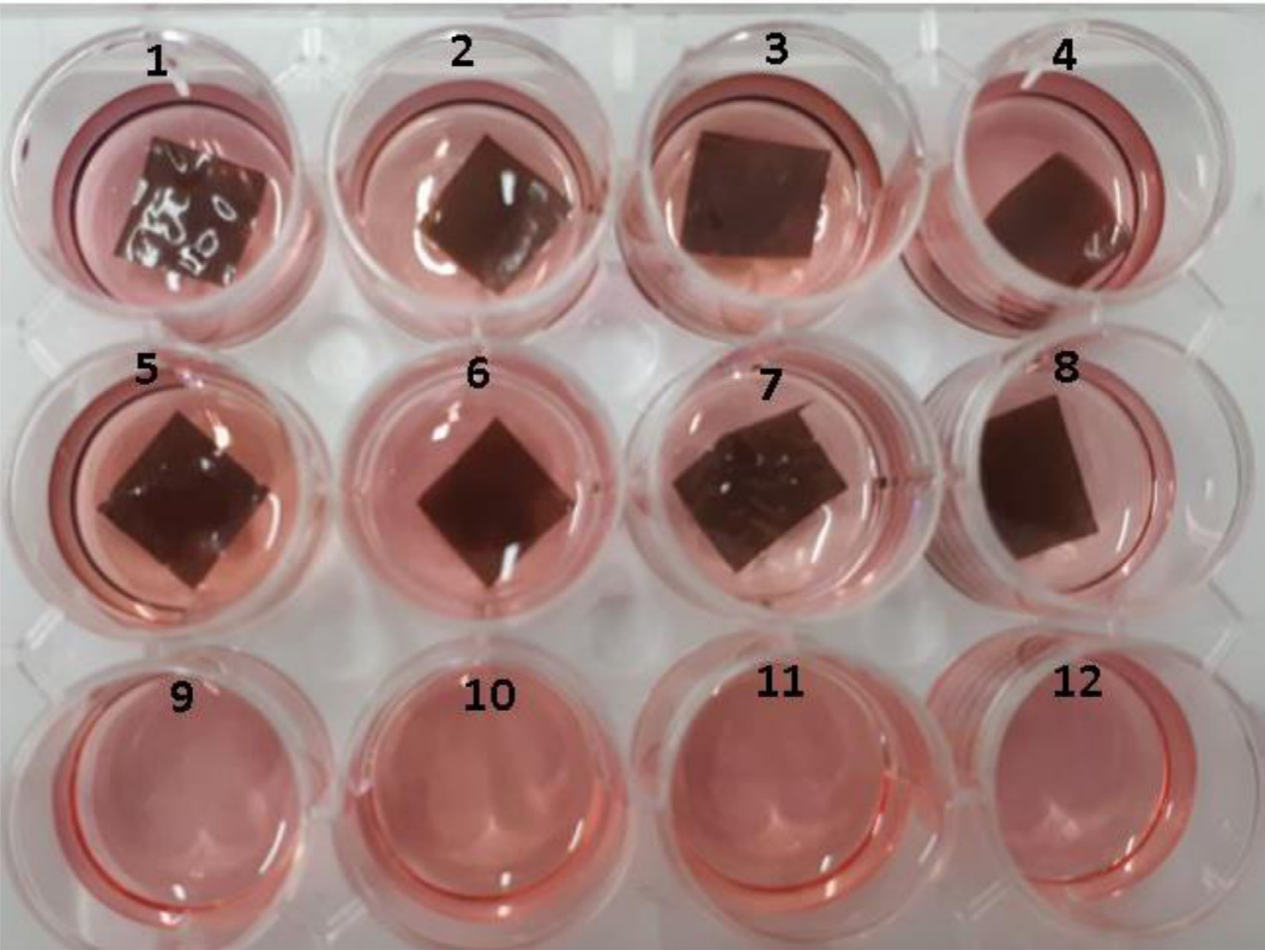
Culture of CS-GO and CS/GO films with gingival fibroblasts. Culture wells 1–4, CS-GO sample. Culture wells 5–8, CS/GO sample and culture well 9–12, control with no samples.

**Fig. 4:**
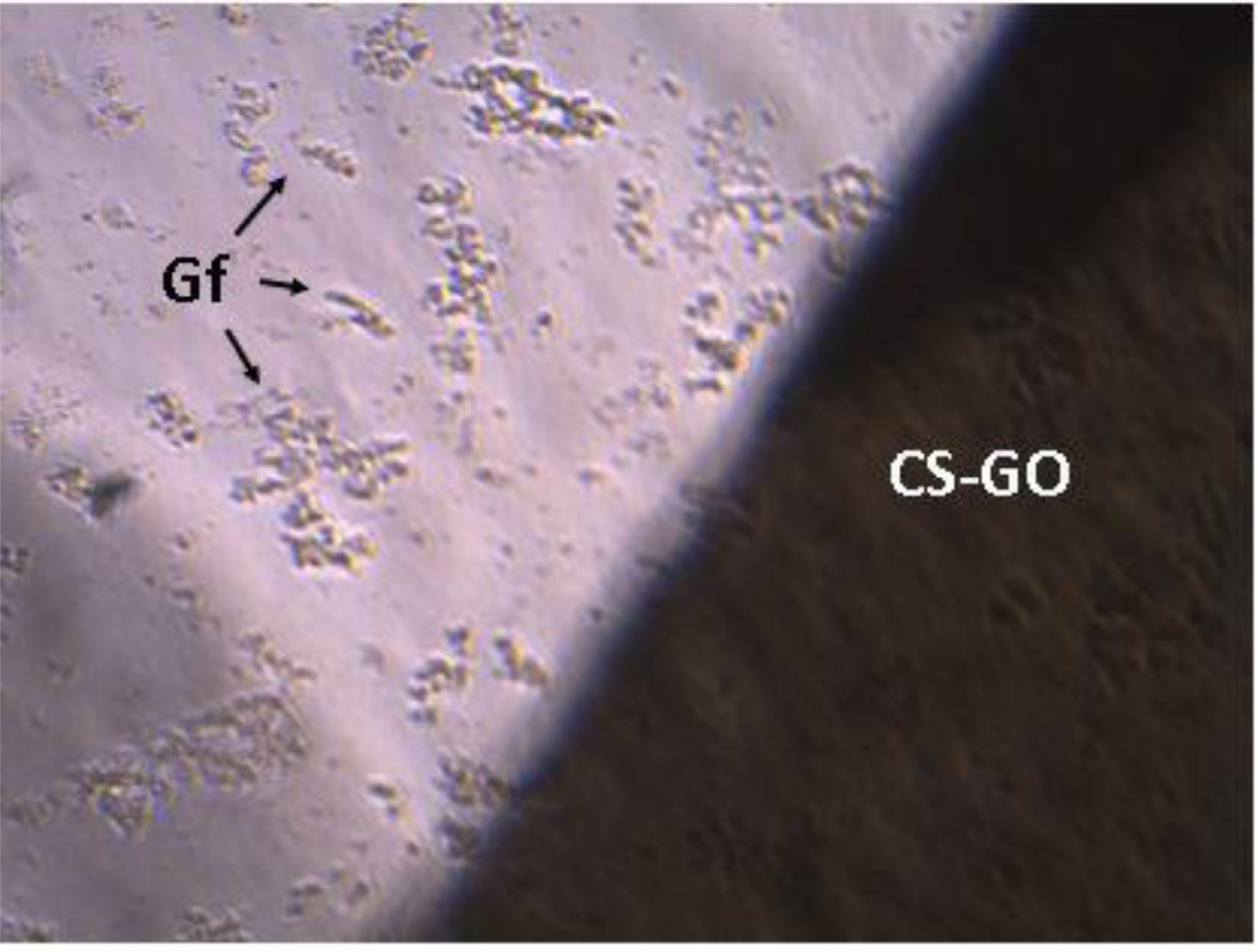
Culture of CS-GO membrane with gingival fibroblasts. Image at 4 × . Gf: Gingival fibroblast, CS-GO: CS-GO film.

For the cell growth test, the gingival fibroblast was cultured into a film of CS-GO (Fig. 3 and Fig. 4), and after nine days, films were taken from the culture well, and cells were detached. A Neubauer camera counted cells, and it was found an average number of 38,000 cells in each well contained in the sample and 40000 cells in control well.

## 2 Experimental Design, Materials and Methods

### 2.1 Materials

For GPC analysis, six pullulan standards were used with a mass average molar mass (Mw) 1320, 6200, 10000, 21700, 201100, and 586800 Da (Sigma-Aldrich, Palo alto, CA, USA). The mobile phase consisted of a solution of NaNO_3_ (Merck KGaA, Darmstadt, Germany) and CH_3_COOH (Sigma-Aldrich, Palo alto, CA, USA) 0.5 M. Chitosan (Sigma-Aldrich, Palo alto, CA, USA) and Graphene Oxide prepared by the Hummer-Offemann modified method previously reported [5], CS-GO was obtained by the functionalization reaction as described elsewhere. The cell growth test used trypsin in PBS (Sigma-AldrichCorp. St. Louis, MO USA), Dubelco Modified Medium (Invitrogen, Carlsbad, CS, USA).

### 2.2 Methods

#### 2.2.1 Chromatographic analysis

The United States Pharmacopeial Convention (USP) guide for the chromatographic analysis was used to determine the molecular weight analysis by GPC. For this analysis, a phase mobile of NaNO_3_ 0.15 M and CH_3_COOH 0.5 M was used, samples of CS and CS-GO were dilute into the mobile phase in a concentration of 1 mg/mL, both samples and the mobile phase was filtered with a PVDF membrane of 0.45 µm.

Measurement was carried out into the Agilent Infinity 1260 with a refractive index detector and two Shodex OHpack 805 HQ and 806 HQ columns.

#### 2.2.2 Biocompatibility test

Biocompatibility test in vitro was performed using brine shrimp, and the Rocha-Filho protocol [1], aeration with a pump was used, and illumination and permanent lighting was maintained with a 110 V and 60 W lamp. After 24 h of hydration, eggs were deposited in a recipe with a dark part and light part, looking for the larvae to emerge once, the most vigorous will swim towards the region illuminated by the phototropic effect.

A box with 24 wells for cell culture was used for this test, films of samples (CS-GO and CS/GO) of 10 × 10 mm were deposited in 12 wells. Later, 1.5 mL of a seawater solution and ten nauplii were added to each of them. As a control, four additional wells were left with 1.5 mL of solution and ten nauplii in each of them, but without samples. Negative control was not used.

#### 2.2.3 Cellular growth test

For the cell growth test, it was necessary to unfrozen and taken to incubation in a New Brunswick Galaxy 48R incubator (Eppendorf, Hauppauge, NY, USA) with complete culture medium Dubelco Modified Medium (DMEM) + 10% Fetal Bovine Serum + Antibiotic / Antifungal. This medium was replaced every three days, and after 15 days, a suitable confluence for the culture procedures with the samples was obtained.

In a box of 12 culture wells deposited eight film samples of 10 × 10 mm, approximately 30,000 cells, and 1 mL of complete culture medium. The medium was changed every three days; the membranes were removed at 9-day, and the cells were detached by trypsinization with 0.25% trypsin in PBS (Sigma-AldrichCorp. St. Louis, MO USA). Subsequently, images were taken using a Leica inverted field microscope (Leica Microsystems, Mannheim, Germany) to verify cell growth, and the number of cells was counted using a Neubauer camera (Blood Counting Chambers, BOECO, Hamburg Germany).

## Ethics Statement

In this research, brine shrimp was used obtained from a local fish farming distributor. Gingival fibroblasts were supplied by the *in-vitro* Cell Culture Laboratory of the Universidad del Valle. None of them have a restriction on its use; this research was authorized by the ethical committee of the Universidad del Valle (CEAS 001-016).

## CRediT authorship contribution statement

**Ana Maria Valencia:** Methodology, Writing – original draft. **Carlos Humberto Valencia:** Conceptualization, Methodology, Writing – original draft. **Fabio Zuluaga:** Writing – review & editing, Supervision. **Carlos David Grande-Tovar:** Visualization, Supervision, Writing – review & editing.

## Declaration of Competing Interest

The authors declare that they have no known competing financial interests or personal relationships which have, or could be perceived to have, influenced the work reported in this article.
